# Comparative FISH mapping of ribosomal DNA clusters and TTAGG telomeric sequences to holokinetic chromosomes of eight species of the insect order Psocoptera

**DOI:** 10.3897/CompCytogen.v13i4.48891

**Published:** 2019-12-06

**Authors:** Natalia Golub, Boris Anokhin, Valentina Kuznetsova

**Affiliations:** 1 Zoological Institute, Russian Academy of Sciences, Universitetskaya emb. 1, St. Petersburg 199034, Russia Zoological Institute, Russian Academy of Sciences St. Petersburg Russia

**Keywords:** Insecta, psocids, Psocomorpha, meiosis, holokinetic chromosomes, (TTAGG)_n_, 18S rDNA, FISH

## Abstract

Repetitive DNAs are the main components of eukaryotic genome. We mapped the 18S rDNA and TTAGG telomeric probe sequences by FISH to meiotic chromosomes of eight species of the order Psocoptera considered a basal taxon of Paraneoptera: *Valenzuela burmeisteri* (Brauer, 1876), *Stenopsocus lachlani* Kolbe, 1960, *Graphopsocus cruciatus* (Linnaeus, 1768), *Peripsocus phaeopterus* (Stephens, 1836), *Philotarsus picicornis* (Fabricius, 1793), *Amphigerontia bifasciata* (Latreille, 1799), *Psococerastis gibbosa* (Sulzer, 1766), and *Metylophorus nebulosus* (Stephens, 1836). These species belong to five distantly related families of the largest psocid suborder Psocomorpha: Caeciliusidae, Stenopsocidae, Peripsocidae, Philotarsidae, and Psocidae. We show that all the examined species share a similar location of 18S rDNA on a medium-sized pair of autosomes. This is the first study of rDNA clusters in the order Psocoptera using FISH. We also demonstrate that these species have the classical insect (TTAGG)_n_ telomere organization. Our results provide a foundation for further cytogenetic characterization and chromosome evolution studies in Psocoptera.

## Introduction

Psocoptera (booklice and barklice) are a small insect order considered a basal taxon of Paraneoptera (Yoshizawa and Saigusa 2001). The order includes 5941 species in 485 genera, 41 families and 3 suborders: Trogiomorpha, Troctomorpha and Psocomorpha (Mockford 2018). To date, 90 psocopteran species (51 genera, 21 families) have been cytogenetically studied, most of them (80 species, 43 genera, 16 families) belonging to the largest suborder Psocomorpha (reviewed in Golub and Nokkala 2009). In this suborder, the majority of species (71 species from 36 genera and 15 families) display 2n = 16 + XX/X(0) indicative of a particular karyotype conservatism of the group. This karyotype is considered modal and ancestral both for Psocomorpha and for the order Psocoptera in general (Wong and Thornton 1966, Golub 1999, Golub and Nokkala 2009). To date, different derived karyotypes have been reported only for nine species: *Amphipsocus japonicus* Enderlein, 1906 and *Kolbia quisquiliarum* Bertkau, 1882 from the family Amphipsocidae (2n = 14 + XX/neo-XY); *Elipsocus moebiusi* Tetens, 1891 and *Loensia variegata* (Latreilee, 1799) from the families Elipsocidae and Psocidae, respectively (2n = 12 +XX/X(0); *Neopsocopsis hitricornis* (Reuter, 1893), *Metylophorus nebulosus* (Stephens, 1836), and *Amphigerontia jezoensis* Okamoto, 1907 from the family Psocidae (2n = 14 +XX/X(0); *Stenopsocus lachlani* Kolbe, 1960 and *Stenopsocus aphidiformis* Enderlein, 1906 from the family Stenopsocidae (2n = 22 +XX/X(0)).

Psocoptera are characterized by holokinetic chromosomes (Meinander et al. 1974, Golub and Nokkala 2009), which are known to lack such physical landmarks as primary constrictions (the centromeres) and, thus, show no distinguishable markers that could be studied by conventional techniques. In the majority of psocid species, the chromosomes are small and of similar size, making it impossible to identify individual chromosomes. Although karyotypes have been described for many psocid species, individual chromosomes were not identified in most of these reported karyotypes. Such chromosome sets, therefore, are not comparable among related species and cannot be used for evolutionary studies.

The application of banding techniques to chromosome studies of Psocoptera is scarce (Golub and Nokkala 2001, Golub et al. 2004). Golub et al. (2004) used C-banding, silver impregnation and sequence-specific fluorochromes CMA_3_ and DAPI to study male meiotic karyotypes of *Psococerastis gibbosa* (Sulzer, 1766) with 2n = 16 + X(0), *Blaste conspurcata* (Rambur, 1842) with 2n = 16 + X(0), and *Amphipsocus japonicus* with 2n = 14 + neo-XY. Based on the results obtained, the authors had concluded that NORs (nucleolus organizer regions) were located differently in these species: on an autosomal bivalent, on the X chromosome, and on the neo-XY bivalent, respectively. We believe however that additional studies are needed to confirm the precise localization of NORs in the above species. Using C-banding, the authors found minor interspecific differences in amount, molecular composition and localization of C-heterochromatin as well as some analogous differences between various chromosomes of a particular species.

Our knowledge of karyotype structure and evolution in Psocoptera could be improved by the implementation of molecular cytogenetic approaches. Fluorescence *in situ* hybridization (FISH) has become the most important technique for tracing individual chromosomes in holokinetic insects (e.g., Panzera et al. 2012, 2015, Maryańska-Nadachowska et al. 2013, 2018, Mandrioli et al. 2014, Kuznetsova et al. 2015, Anjos et al. 2016, Golub et al. 2017, Salanitro et al. 2017, Grozeva et al. 2019). It was shown in some case studies that species with the same chromosome complement differ in the number and location of rDNA sites (Panzera et al. 2012, 2015, Maryańska-Nadachowska et al. 2013, Golub et al. 2017). Moreover, some higher insect taxa were shown to differ in respect to the presence/absence of the insect-type telomere motif (TTAGG)_n_. Specifically, such variation has been demonstrated for some Paraneoptera, e.g. Hemiptera, where more basal taxa appear to have the ancestral insect telomere motif (TTAGG)_n_ while more advanced taxa have lost this telomeric sequence (reviewed in Kuznetsova et al. 2019). The only psocid species studied so far by FISH, *Stenopsocus lachlani* (Psocomorpha, Stenopsocidae), was documented to have the (TTAGG)_n_ telomere motif (Frydrychová et al. 2004).

Here, we used FISH with the telomeric TTAGG and 18S rDNA probes to study male meiotic chromosomes of *Valenzuela burmeisteri* (Brauer, 1876), *Stenopsocus lachlani*, *Graphopsocus cruciatus* (Linnaeus, 1768), *Peripsocus phaeopterus* (Stephens, 1836), *Philotarsus picicornis* (Fabricius, 1793), *Amphigerontia bifasciata* (Latreille, 1799), *Psococerastis gibbosa*, and *Metylophorus nebulosus*. The standard karyotypes of these species were previously reported (reviewed in Golub and Nokkala 2009). We demonstrate that the above species, belonging to five different families of the largest suborder Psocomorpha (Caeciliusidae, Stenopsocidae, Peripsocidae, Philotarsidae, and Psocidae), are characterized by conserved karyotypes in respect to telomere composition and rDNA location. This is the first study of rDNA clusters in the order Psocoptera using FISH.

## Material and methods

The information on the localities where the specimens were collected and on the number of specimens/nuclei examined is presented in Table 1. Only male adult specimens were analyzed. Males were fixed in the Carnoy fixative (3:1; 96% ethanol and glacial acetic acid) and stored at 4 °C. Testes were dissected out in a drop of 45% acetic acid and squashed. The cover slips were removed using dry ice. Prior to staining, the preparations were examined by phase contrast microscopy.

**Table 1.** Material studied.

Species	Collection date and localities	Number of studied males / nuclei
Fam. Caeciliusidae		
*Valenzuela burmeisteri*	Russia, the Altai Republic, Artybash vic., 51°47'28"N, 87°15'21"W, July, 2019	4/12
Fam. Stenopsocidae		
*Stenopsocus lachlani*	Russia, the Altai Republic, Artybash vic., 51°47'28"N, 87°15'21"W, July, 2019	2/22
*Graphopsocus cruciatus*	Russia, Voronezh region, Maklok vic., 51°48'42"N, 39°24'51"W, August, 2018	4/18
Fam. Peripsocidae		
*Peripsocus phaeopterus*	Russia, the Altai Republic, Artybash vic., 51°47'28"N, 87°15'21"W, July, 2019	6/30
Fam. Philotarsidae		
*Philotarsus picicornis*	Russia, the Altai Republic, Artybash vic., 51°47'28"N, 87°15'21"W, July, 2019	6/42
Fam. Psocidae		
*Amphigerontia bifasciata*	Russia, Karachay-Cherkess Republic, Teberda vic., 43°27'00"N, 41°45'00"W, July, 2017	2/10
*Metylophorus nebulosus*	Russia, the Altai Republic, Artybash vic., 51°47'28"N, 87°15'21"W, July, 2019	5/28
*Psococerastis gibbosa*	Russia, the Altai Republic, Artybash vic., 51°47'28"N, 87°15'21"W, July, 2019	4/46

Fluorescence in situ hybridization was performed according to the published protocol (Grozeva et al. 2015) with minor modifications. The target 18S rDNA probe (about 1200 bp fragment) was PCR amplified and labelled with biotin-11-dUTP (Fermentas, EU) using primers: 18SrRNA_F 5’-GATCCTGCCAGTAGTCATATG-3’, 18SrRNA_R 5’-GAGTCAAATTAAGCCGCAGG-3’ (Anokhin et al. 2010). Genomic DNA was extracted from the true bug *Pyrrhocoris apterus* (Linnaeus, 1758). An initial denaturation period of 3 min at 94 °C was followed by 35 cycles of 30 s at 94 °C, annealing for 30 s at 55.5 °C and extension for 1.5 min at 72 °C, with a final extension step of 3 min at 72 °C. The telomere probe (TTAGG)_n_ was amplified by PCR and labeled with rhodamine-5-dUTP (GeneCraft, Köln, Germany) using primers: TTAGG_F 5’-TAACCTAACCTAACCTAACCTAA-3’ and TTAGG_R 5’-GGTTAGGTTAGGTTAGGTTAGG-3’ (Grozeva et al. 2011). An initial denaturation period of 3 min at 94 °C was followed by 30 cycles of 45 s at 94 °C, annealing for 30 s at 50 °C and extension for 50 s at 72 °C, with a final extension step of 3 min at 72 °C. The chromosome preparations were treated with 100 μg/ml RNase A and 5 mg/ml pepsin solution to remove excess RNA and proteins. Chromosomes were denatured in the hybridization mixture containing labelled 18S rDNA and (TTAGG)_n_ probes with an addition of salmon sperm blocking reagent and then hybridized for 42 h at 37 °C. 18S rDNA probes were detected with NeutrAvidin-Fluorescein conjugate (Invitrogen, Karlsbad, CA, USA). The chromosomes were mounted in an antifade medium (ProLong Gold antifade reagent with DAPI, Invitrogen) and covered with a glass coverslip.

## Results and discussion

### Standard karyotypes

All chromosome numbers fully correspond to the previously published karyotype data for all studied species (reviewed in Golub and Nokkala 2009). Males of *Valenzuela burmeisteri*, *Graphopsocus cruciatus*, *Peripsocus phaeopterus*, *Philotarsus picicornis*, *Amphigerontia bifasciata*, and *Psococerastis gibbosa* were confirmed to have 2n = 16 + X(0), the chromosome complement known to be the most characteristic and presumably ancestral for Psocoptera (Wong and Thornton 1966, Golub 1999, Golub and Nokkala 2009). Males of *Stenopsocus lachlani* and *Metylophorus nebulosus* were confirmed to have 2n = 22 + X(0) and 2n = 14 + X(0), respectively. Thus, even though the sex chromosome system is the same among all studied species, the number of autosomes differs considerably between them. Based on the meiotic figures, we can infer that the karyotype structure of these species is uniform: all the bivalents constitute a decreasing size series, which makes identifying individual bivalents almost impossible. The only exception to this rule is the karyotype of *M. nebulosus*. In this species, metaphase I nuclei were shown to include seven bivalents with a particular element being significantly larger than the other ones (Meinander et al. 1974, Golub 1999, present study).

### FISH mapping of 18S rDNA repeats

**Figure 1.**
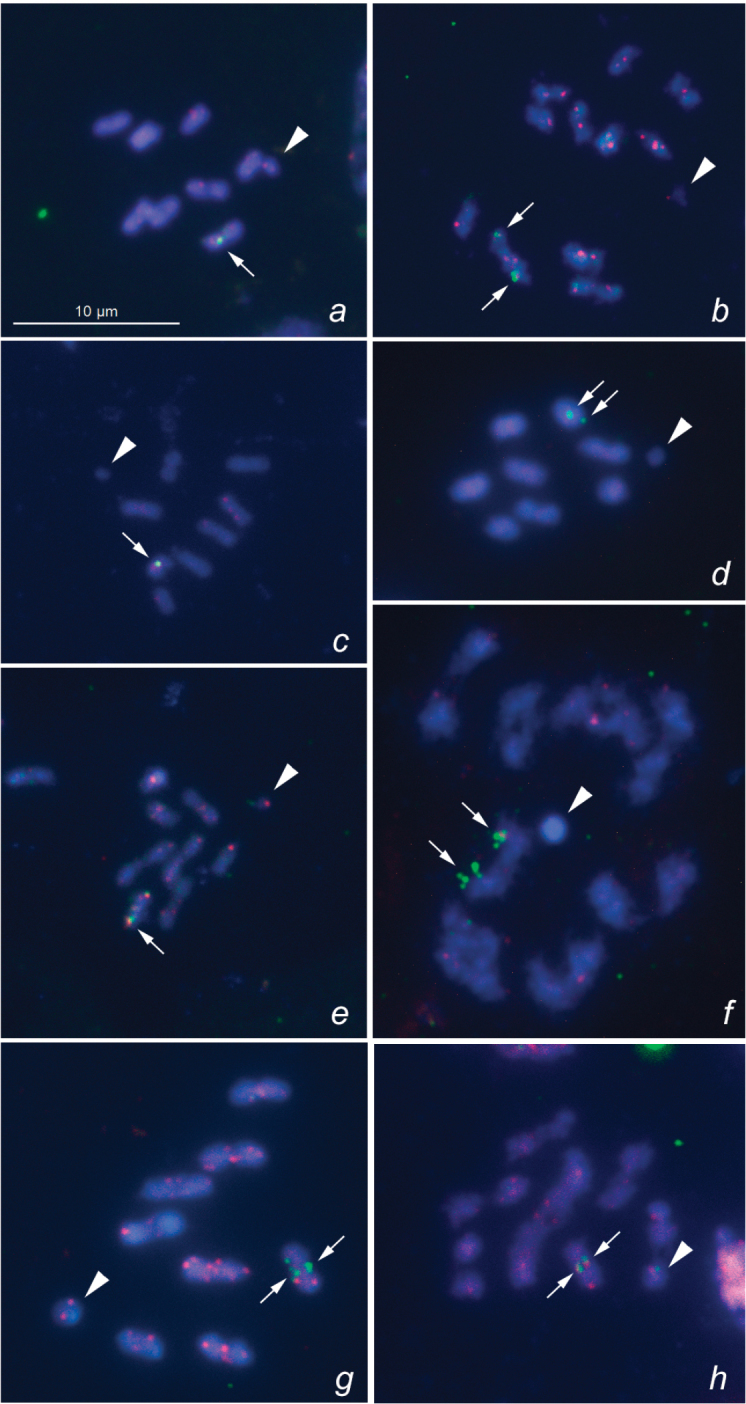
FISH mapping of TTAGG telomeric sequences (red signals) and 18S rDNA (green signals) to meiotic chromosomes of Psocoptera**a***Valenzuela burmeisteri*, MI, n = 8 + X **b***Stenopsocus lachlani*, MI, n = 11 + X **c***Graphopsocus cruciatus*, MI, n = 8 + X **d***Peripsocus phaeopterus*, MI, n = 8 + X **e***Philotarsus picicornis*, MI, n = 8 + X **f***Amphigerontia bifasciata*, diakinesis, n = 8 + X **g***Psococerastis gibbosa*, MI, n = 8 + X **h***Metylophorus nebulosus*, MI, n = 7 + X. Arrowheads and arrows indicate sex chromosomes and 18S rDNA signals, respectively. Scale bar: 10 μm.

In each of the species studied, FISH mapping with the 18S rDNA probe revealed two large clusters located in a sub-terminal position on the homologues of a medium-sized bivalent (Fig. 1a–h). The signals could be observed either in the chiasmate or the opposite region of the rDNA-carrying bivalent, thus suggesting that the same homologue is able to orient differently within the bivalent. Following current knowledge, all the studied species are suggested to share a similar chromosomal location of the rRNA genes on the same pair of autosomes. However, this speculation is premature, since the precise identification of particular bivalents in psocid karyotypes is currently impossible due to the absence of additional differential chromosomal landmarks.

### FISH mapping of TTAGG telomeric repeats

In each of the species studied, FISH mapping with TTAGG repeats revealed signals located in a telomeric position on the chromosomes. The signals were visible in most but not all terminal regions of meiotic chromosomes. Moreover, in some species, the signals were bright (Fig. 1b, e, g, h), whereas in other species they were not so clearly defined (Fig. 1a, c, d, f).

A previous investigation by Frydrychová et al. (2004) documented presence of the (TTAGG)_n_ telomere motif in *S. lachlani* (Stenopsocidae). Despite the variability in the signal intensity, the currently existing data on eight genera from five different families lead to the conclusion that psocids, at least those from the suborder Psocomorpha, share the telomere structure (TTAGG)_n_ known to be characteristic of the majority of insect orders and considered ancestral for the class Insecta in general (Kuznetsova et al. 2019).

In conclusion, the present study contributes to the understanding of the chromosome structure of Psocoptera and provides a foundation for further cytogenetic characterization and chromosome evolution studies in this group.
